# The complete chloroplast genome of *Leptochilus hemionitideus*, a traditional Chinese medical fern

**DOI:** 10.1080/23802359.2018.1491345

**Published:** 2018-07-13

**Authors:** Yihui Min, Jingyi Guan, Shufeng Li, Shanshan Liu, Yongfeng Hong, Zhen Wang, Ting Wang, Yingjuan Su

**Affiliations:** a School of Life Sciences, Sun Yat-sen University, Guangzhou, China;; b College of Life Sciences, Nanjing Agricultural University, Nanjing, China;; c College of Life Sciences, South China Agricultural University, Guangzhou, China;; d Research Institute of Sun Yat-sen University in Shenzhen, Shenzhen, China

**Keywords:** *Leptochilus hemionitideus*, chloroplast genome, phylogenetic analysis

## Abstract

The complete chloroplast genome of *Leptochilus hemionitideus* was sequenced. Its length is 156,083 bp with 44.2% GC content. The genome exhibits typical quadripartite with two inverted repeat regions (24,594 bp, each) separated by a large single-copy (LSC, 81,403 bp) region and a small single-copy (SSC, 25,492 bp) region. It has 131 genes, including 87 protein-coding genes, 34 tRNA genes, eight rRNA genes and two pseudogenes. Maximum-likelihood phylogenetic tree indicated that *L. hemionitideus* was closely related to *Lepisorus clathratus*. The complete chloroplast genome of *L. hemionitideus* would provide very valuable molecular information for further inferring the relationships of the microsoroid ferns.

*Leptochilus hemionitideus* is terrestrial fern belonging to the subfamily Microsoroideae in Polypodiaceae (Zhang et al. 2013). Different from congeneric other species, it has orbicular to elongate sori on tertiary veins parallel to secondary veins (Zhang et al. 2013). Its fronds are not or only slightly dimorphic. The plant has rock habit, usually growing on stones in streams at an altitude of 700–2000 m (Zhang et al. 2013). Its main distribution areas are concentrated in China, Bhutan, India, Japan, Nepal and Thailand (Zhang et al. 2013). In China, *L. hemionitideus* is a traditional Chinese medical fern with clearing heat and detoxification (State Administration of Traditional Chinese Medicine ‘Chinese Materia Medica Committee’ 1999). In addition, whether *Leptochilus* were merged with *Colysis* occurs controversy due to lack of the obvious generic delimitation (Nooteboom 1997; Shi and Zhang 1999). Phylogenetic relationships of *Leptochilus* to some genera in Microsoroideae such as *Microsorum* are also needed to further explore (Christenhusz and Chase 2014; PPG I 2016). Therefore, sequencing complete chloroplast genome of *L. hemionitideus* will contribute to deal with these issues and lay solid foundations for further phylogenomic investigation.

We sampled fresh and young leaves of *L. hemionitideus* from South China Botanical Garden, Chinese Academy of Sciences (CAS; 23°11′3.56″N, 113°21′43.28″E). The voucher specimen was conserved in Herbarium of Sun Yat-sen University (SYS; voucher: *SS Liu 20161014*). After total genomic DNA extraction, we built up ∼300 bp genomic library and sequenced in Illumina Hiseq 2500 platform (Illumina Inc., San Diego, CA). Total 8,119,002 raw reads were retrieved and trimmed by Trimmomatic (Bolger et al. 2014). A subset of 6,801,257 trimmed reads was used for reconstructing the chloroplast genome by Velvet v1.2.07 (Zerbino and Birney 2008) with 33× coverage. The chloroplast genome was annotated using DOGMA (Wyman et al. 2004) and tRNAscan-SE (Schattner et al. 2005) programs with default settings and finally validated with BLAST searches and manually corrected for intron/exon boundaries. The complete chloroplast genome sequence of *L. hemionitideus* was aligned with 11 representative ferns including *Marsilea crenata* as outgroup using MAFFT v7.311 (Katoh and Standley 2013). A maximum likelihood (ML) phylogenetic tree was constructed using RAxML v.8.0 with 1000 bootstrap replicates (Stamatakis 2014).

We determined complete chloroplast genome of *L. hemionitideus*, which possesses a total length of 156,083 bp with 44.2% GC content (GenBank accession number: MH319943). The circular cp genome exhibits typical quadripartite with two inverted repeat regions (IRa and IRb) of 24,594 bp separated by a large single-copy (LSC) region of 81,403 bp and a small single-copy (SSC) region of 25,492 bp. It was predicted to contain 131 genes, including 87 protein-coding genes, 34 tRNA genes, eight rRNA genes and two pseudogenes (*ndh*B and *rpo*C1). Among them, 115 genes occur as a single copy, whereas 14 genes are duplicated in the IR regions. Fourteen genes contain one intron, especially, the gene *ycf*3, *clp*P, and *rps*12 have two introns. ML tree indicated that *L. hemionitideus* is closely related to *Lepisorus clathratus* (Figure 1). The complete chloroplast genome of *L. hemionitideus* will provide very valuable molecular information for further inferring the relationships of the microsoroid ferns.

**Figure 1.**
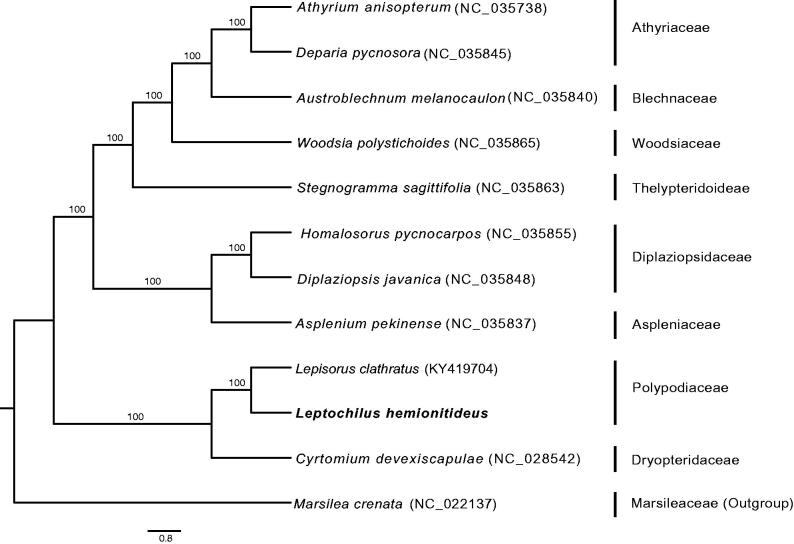
ML phylogenetic tree inferred based on complete chloroplast genomes of 12 representative ferns including *Marsilea crenata* as outgroup. Node labels indicate the bootstrap support values with 1000 replicates.
